# Genetic diversity and population structure of wheat landraces in Southern Winter Wheat Region of China

**DOI:** 10.1186/s12864-024-10564-z

**Published:** 2024-07-03

**Authors:** Ying Liu, Bisheng Fu, Qiaofeng Zhang, Jin Cai, Wei Guo, Wenling Zhai, Jizhong Wu

**Affiliations:** 1 Institute of Germplasm Resources and Biotechnology/Jiangsu Provincial Key Laboratory of Agrobiology, Jiangsu Academy of Agricultural Sciences, Nanjing, Jiangsu 210014 China; 2 Zhongshan Biological Breeding Laboratory, Nanjing, Jiangsu 210014 China; 3 Jiangsu Co-Innovation Center for Modern Production Technology of Grain Crops, Yangzhou University, Yangzhou, 225009 China

**Keywords:** *Triticum aestivum.* L, Landrace, Core collection, Genetic diversity, Population structure

## Abstract

**Background:**

Wheat landraces are considered a valuable source of genetic diversity for breeding programs. It is useful to evaluate the genetic diversity in breeding studies such as marker-assisted selection (MAS), genome-wide association studies (GWAS), and genomic selection. In addition, constructing a core germplasm set that represents the genetic diversity of the entire variety set is of great significance for the efficient conservation and utilization of wheat landrace germplasms.

**Results:**

To understand the genetic diversity in wheat landrace, 2,023 accessions in the Jiangsu Provincial Crop Germplasm Resource Bank were used to explore the molecular diversity and population structure using the Illumina 15 K single nucleotide polymorphism (SNP) chip. These accessions were divided into five subpopulations based on population structure, principal coordinate and kinship analysis. A significant variation was found within and among the subpopulations based on the molecular variance analysis (AMOVA). Subpopulation 3 showed more genetic variability based on the different allelic patterns (Na, Ne and I). The M strategy as implemented in MStratv 4.1 software was used to construct the representative core collection. A core collection with a total of 311 accessions (15.37%) was selected from the entire landrace germplasm based on genotype and 12 different phenotypic traits. Compared to the initial landrace collections, the core collection displayed higher gene diversity (0.31) and polymorphism information content (PIC) (0.25), and represented almost all phenotypic variation.

**Conclusions:**

A core collection comprising 311 accessions containing 100% of the genetic variation in the initial population was developed. This collection provides a germplasm base for effective management, conservation, and utilization of the variation in the original set.

**Supplementary Information:**

The online version contains supplementary material available at 10.1186/s12864-024-10564-z.

## Background

Wheat is one of the most important staple crops for more than one-third of the human population, providing about 19% of the calories and 21% of the protein [1]. Approximately 90 to 95% of wheat grown worldwide is bread wheat (*Triticum aestivum* L.) (2n = 6x = 42, AABBDD) [2]. Multiple rounds of rare natural hybridization between different wheat species and relatives led to the currently cultivated wheat, but also caused genetic bottlenecks due to the exclusion of adaptive alleles [3, 4]. Modern cultural practices and improved cultivars to take advantage of those practices significantly increased wheat production. However, the development of high-yielding modern wheat cultivars is at the expense of losing much of the diversity in landraces and older varieties. In the last century, wheat landraces were almost completely replaced by modern cultivars, reducing the overall diversity of the species [5].

Wheat landraces show a much higher genetic diversity than elite varieties [6]. Potentially valuable traits in landraces include early growth vigour [7], cold, heat or drought tolerance [8–10], disease resistance, water use efficiency [11], and quality traits suited for local food preferences. Developing new cultivars from landrace populations is a feasible strategy to improve wheat productivity and stability, especially in vulnerable environments in breeding programs.

Scientists have been conscientious in conserving wheat landraces for a long time. Large numbers of landraces were collected, conserved, studied, and analyzed, and the potential for utilization and incorporation of their beneficial traits into new varieties was explored [5]. The Türkiye scientist Gökgöl, collected and characterized 18,000 wheat landraces from Türkiye; among them, 256 varieties were new [12]. More than 60 distinct wheat landraces were collected in five mountainous regions of Tajikistan [13]. Over 30 bread wheat landraces in three regions from the western Tian-Shan mountains were collected in Uzbekistan [14]. These landraces were thoroughly phenotyped, genotyped, conserved in gene banks and used in wheat breeding. In China, nearly 13,900 wheat landraces from different geographic and climatic conditions are conserved in the National Gene Bank [15]. Chinese wheat landraces are characterized by earliness, large numbers of grains per spike, high adaptiveness, and a long history of cultivation [16].

Three strategies were applied to represent and exploit the diversity of landraces in previous studies: (1) measuring diversity and developing a core collection from extensive collections to represent the overall genetic diversity with minimal repetition; (2) exploiting the most favorable alleles of important traits in breeding programs; and (3) retaining phenotypic variation and related genetic association for targeted traits through large-scale and precise phenotypic analysis combined with GWAS [17]. According to Frankel et al. [18], a core collection with the minimum redundancies represents the genetic variation of an entire collection, and facilitates maintenance, research, and utilization of germplasm resources.

During the last few decades, several core collections of wheat have been constructed, and they have played an important role in the conservation and improved use of wheat genetic resources. A worldwide bread wheat core collection of 372 accessions (372CC) was selected with a set of 38 simple sequence repeat (SSR) markers [19]. Hao et al. [20] established a mini-core collection of 231 Chinese wheat accessions with an estimated 70% representation of the genetic variation from the initial collection using 78 SSR markers. Using 36,720 SNP markers, Mourad et al. [21] analyzed the genetic diversity and population structure of a 103 accessions spring wheat core collection representing worldwide germplasm collection.

Wheat is grown in ten agro-ecological zones in China, which vary widely in climate, soil, cultivar adaptation and management. The adaptation to these different environments led to the creation of landraces. China has rich genetic resources of wheat landraces, which are important for production and breeding. In this study, the morphological description and genomic characterization of wheat landraces collected from 2008 to 2014 at the Jiangsu Academy of Agricultural Sciences, Nanjing, China, were undertake to develop opportunities for their use in breeding. In total, 2023 wheat landraces collected from 23 administrative districts were evaluated for agronomic traits in field trials. The genetic diversity was analyzed in a large collection consisting of 2,023 wheat landraces using 15 K Illumina chip. Analyses of the polymorphic markers provided kinship information among groups, the population structure of the accessions, and the genetic properties among subpopulations. We also established a core collection to reduce redundancy in the collection. This core collection will be useful for further utilization of this large set of landraces.

## Methods

### Plant material

**Fig. 1:**
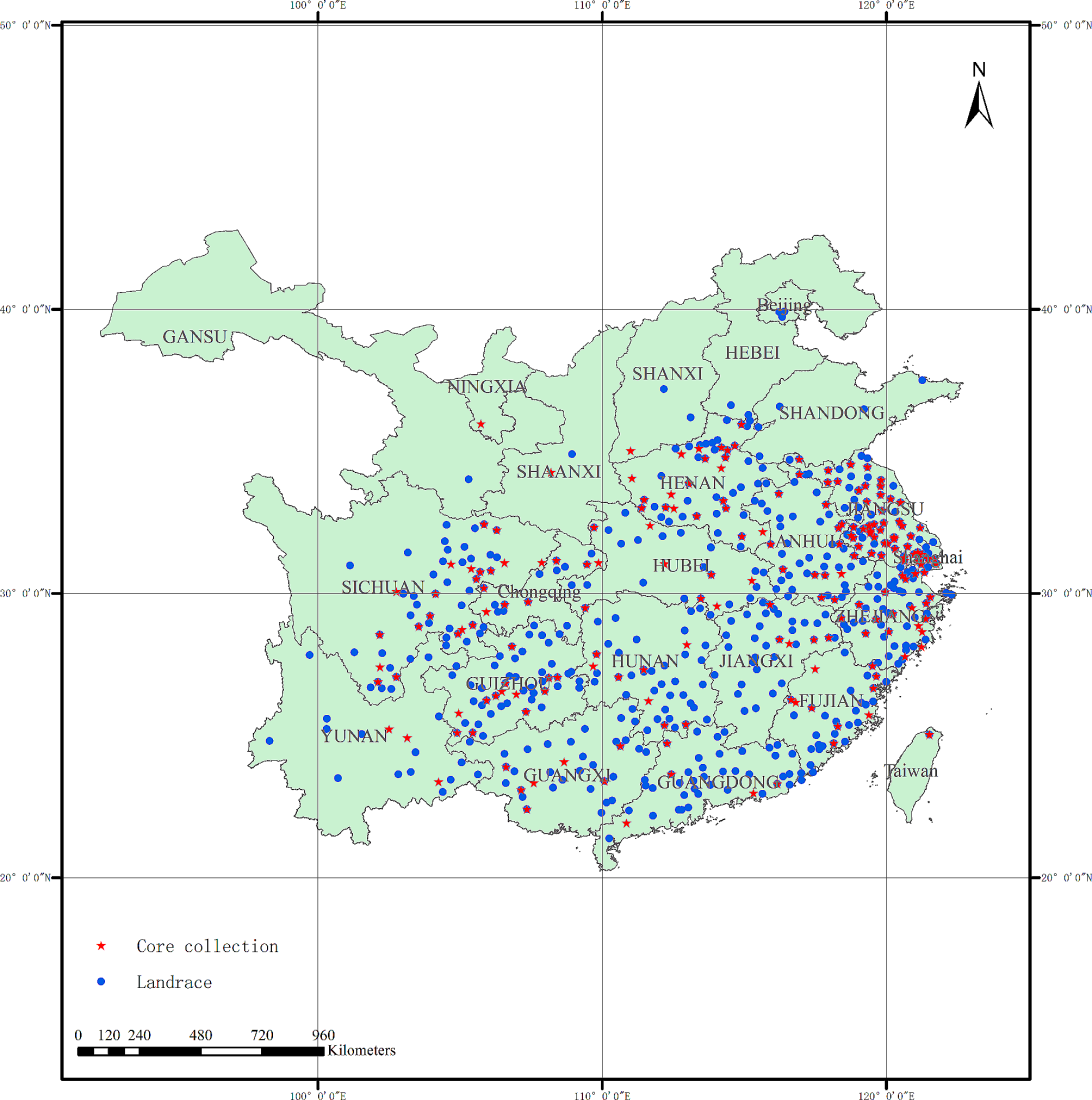
Geographic locations of 2,023 wheat landraces. Red stars represent the geographic distribution of the core collection (311 accessions), and blue dots represent the geographic distribution of the other 1,712 accessions. The core collection is a subset of the original set (2,023 accessions)

We used 2,023 wheat landraces accessions conserved at the Gene Bank, located at the Institute of Germplasm Resources and Biotechnology, Jiangsu Academy of Agricultural Sciences, Nanjing, China. These accessions were collected from 23 provinces in China (Fig. 1). All details about the 2,023 wheat landrace accessions are shown in Additional file 1: Table S1. Of these, 937 (46.32%) accessions were obtained from Jiangsu Province. All of these accessions were precisely evaluated their traits in field trials.

### Phenotyping and data analysis

The 2,023 wheat landraces accessions for twelve agronomics traits were evaluated in two environments, of which 1,526 were evaluated in Luhe in 2018 and 497 in Luhe in 2019, respectively. These traits include heading date and flowering date related to maturity; awn type, glume color, spike type, plant height and spike length in relation to plant morphology; and spikelet number per spike, sterile spikelet number per spike, grain number per spikelet, grain number per spike and thousand kernel weight related to yield. A brief description of each trait and data scoring is presented in Additional file 2: Table. S2. The phenotypic diversity (${H}^{{\prime }}$) was calculated as the Shannon index, ${H}^{{\prime }}={\sum }_{i=1}^{n}{P}_{j}ln{P}_{j}$, where $n$ is the number of phenotypic classes for a character and ${P}_{j}$ is the proportion of the total number of entries in the $i$ class [22]. ${H}^{{\prime }}$ was estimated for the twelve agronomics traits.

### SNP genotyping

DNA samples were genotyped with 15 K Axiom^®^ Wheat Breeder Genotyping Array (China Golden Marker Biotechnology Co., Ltd, Beijing) according to the manufacturer’s guidelines. The array comprised 13,947 SNP markers. Quality filtration was performed on the markers using PLINK v1.07 [23]. Minor allele frequency (MAF) less than 5% (--maf 0.05), individuals with more than 20% missing SNP calls (--mind 0.2) and markers with more than 5% missing data (--geno 0.05) were considered for filtration. Physical map positions of all SNP markers were obtained from the Ensembl plants *Triticum aestivum* database (https://plants.ensembl.org/Triticum_aestivum). Markers lacking information for consensus chromosome location were removed. Finally, 7,926 SNP markers and 2,023 genotypes were subjected to further analysis.

### Analysis of genetic diversity

Parameters measuring the genetic diversity of the population such as PIC, gene diversity, heterozygosity (*H*) and MAF were calculated using PowerMarker V3.25 [24]. Other parameters such as average pairwise divergence or observed nucleotide diversity (π), expected nucleotide diversity or estimated mutation rate (θ) [25] and Tajima’s D [26] were calculated using TASSEL v5.2.65 [27].

The AMOVA and estimation of genetic indices were performed using GenAlex 6.41. For this analysis, the genetic indices such as fixation index (F_ST_), different alleles (Na), number of effective alleles (Ne), Shannon’s index (I), observed heterozygosity (H_O_), expected heterozygosity (H_E_), and inbreeding coefficient (F) were calculated.

### Inference of structure, PCA and kinship

To determine the population structure, a filtered marker set (7,926) was pruned using the linkage disequilibrium (LD) based pruning method in PLINK (--indep-pairwise 10 5 0.3). Population structure analysis was calculated using a Bayesian model-based clustering method with STUCTURE 2.3.4 [28] using the pruned markers (2,228). STRUCTURE was run under the ‘admixture model’ with a burn-in period of 100 000 followed by 100,000 replications of Markov Chain Monte Carlo. Three independent runs each were performed with the number of clusters (K) varying from 1 to 10. The most likely number of subpopulations (K) was determined by using web-based STRUCTURE HARVESTER, and a ΔK statistic based on the relative rate of change in the likelihood of the data between successive K values was used to determine the optimal number of clusters [29, 30]. CLUMPP software was used to generate a consolidated population (Q) matrix from the STRUCTURE runs for the best K value. Lines with probability of membership 0.6 were assigned to a subgroup. Pairwise genetic distances were calculated using the Powermarker V3.25 under the Nei (1983) [31] model. PCA was performed using TASSEL on 7,926 SNP markers. A relative kinship matrix was constructed by TASSEL 5.0, and a heat map was generated in R (http://www.r-project.org) [27]. The geographic structure of the population was studied through PCA and performed on the correlation matrix calculated with the mean country data across years for landraces and the mean data across years for modern cultivars.

### Construction of the core collection

The core collection’s minimal size was estimated using MStrat Software v4.1 [32]. The analysis included three replicates with 30 iterations for each replicate and step of 1 were used. The core collection size was determined based on maximization (M) and random (R) algorithm methods.

## Results

### Genetic diversity of the landrace germplasm

**Fig. 2:**
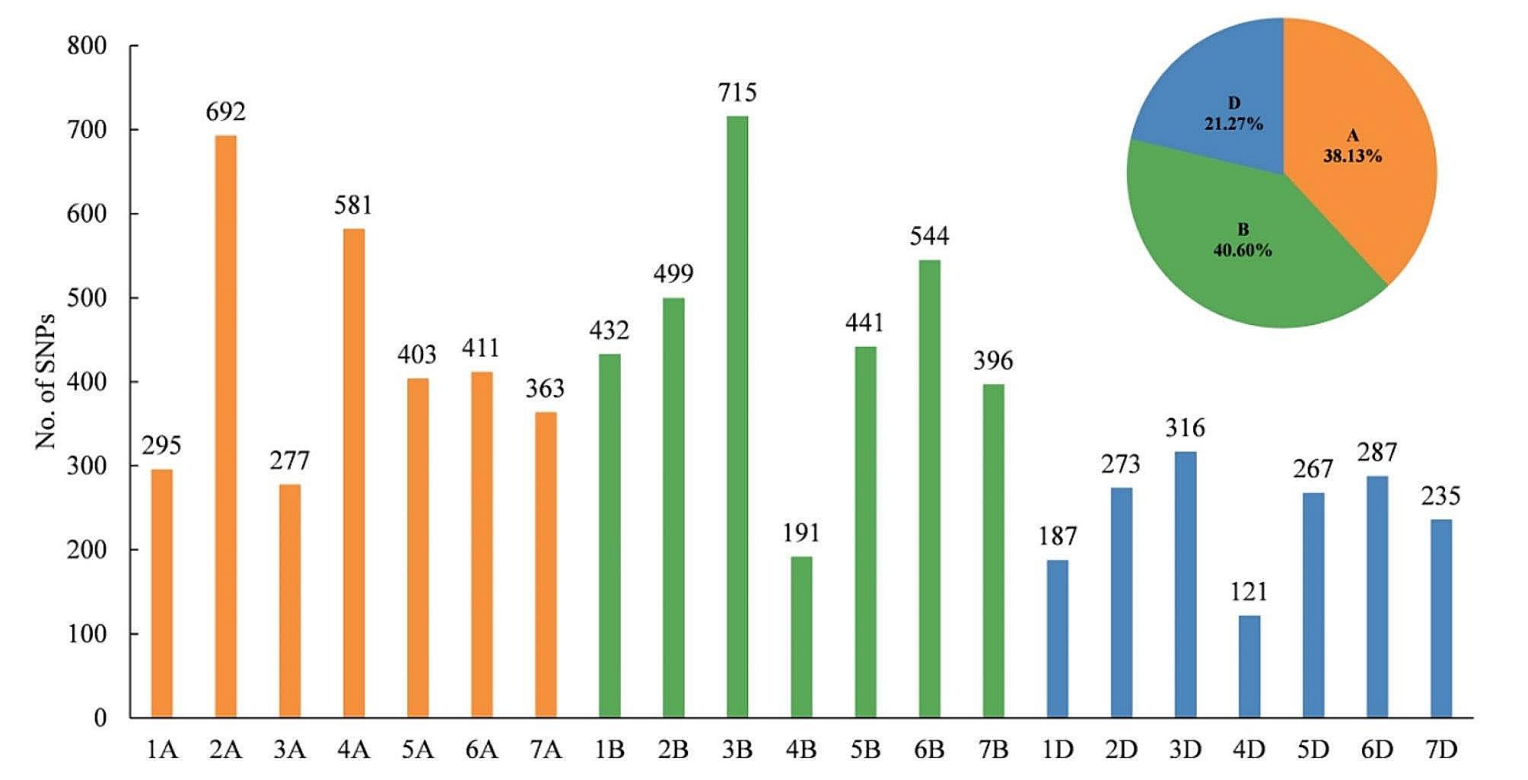
Distribution of 7,963 SNPs across 21 chromosomes of 2,023 wheat landraces

The total number of putative SNPs called from 2,023 wheat landraces were 13,199. After filtering, 7,926 SNP markers were used for genetic diversity, and population structure analysis. The B genome had the highest number of SNPs (3,218, ∼ 40.60%), followed by the A genome (3,022, ∼ 38.13%), and the D genome (1,686, ∼ 21.27%) (Fig. 2; Table 1). The number of SNPs per chromosome ranged from 121 to 715 with an average of 377. In the A genome, chromosome 2A had the highest number of polymorphic markers with 692, and chromosome 3A harbored the lowest number (277); in the B genome, the highest and lowest number of markers were detected on chromosome 3B and 4B (715 and 191, respectively); in the D genome, chromosome 4D had the lowest number of SNPs (121), and chromosome 3D had the highest number (316). To characterize the distribution of SNPs in more detail, we used 1 Mb as a step to plot the distribution of SNPs on each chromosome (Additional file 3: Fig. S1). The number of SNPs on each chromosome was consistent with the physical length of the respective chromosome. The average marker density was approximately 1.77 Mb/SNP. The D genome had the lowest SNP marker density (2.34 Mb/SNP), and the B genome had the highest marker density (1.61 Mb/SNP) (Table 1).

**Table 1:** Summary of genetic diversity among 2,023 landraces accessions. The parameters include number of SNP marker (N), marker coverage, minor allele frequency (MAF), genetic diversity (Hs), heterozygosity(H), polymorphic information content (PIC), nucleotide diversity (π/bp), expected nucleotide diversity (θ/bp) and Tajima’s D

Chr	*N*	Marker coverage (Mb)	MAF	Hs	H	PIC	π/bp	θ/bp	Tajima’s D
1A	295	2.01	0.80	0.27	0.04	0.22	0.27	0.12	3.43
2A	692	1.13	0.79	0.31	0.03	0.26	0.31	0.12	4.36
3A	277	2.71	0.85	0.23	0.03	0.20	0.35	0.12	5.24
4A	581	1.27	0.81	0.29	0.03	0.24	0.32	0.12	4.46
5A	403	1.76	0.82	0.26	0.03	0.22	0.30	0.12	4.03
6A	411	1.50	0.74	0.36	0.04	0.29	0.31	0.12	4.39
7A	363	2.03	0.82	0.26	0.04	0.21	0.23	0.12	2.50
1B	432	1.59	0.77	0.31	0.04	0.25	0.27	0.12	3.43
2B	499	1.60	0.79	0.30	0.04	0.24	0.33	0.12	4.68
3B	715	1.16	0.82	0.27	0.03	0.23	0.29	0.12	3.77
4B	191	3.52	0.77	0.31	0.06	0.25	0.31	0.12	4.31
5B	441	1.62	0.82	0.26	0.04	0.22	0.31	0.12	4.19
6B	544	1.32	0.77	0.33	0.04	0.27	0.21	0.12	3.19
7B	396	1.89	0.77	0.31	0.05	0.25	0.26	0.12	3.12
1D	187	2.65	0.72	0.36	0.06	0.28	0.29	0.12	3.74
2D	273	2.38	0.78	0.32	0.08	0.26	0.36	0.12	5.51
3D	316	1.95	0.77	0.33	0.05	0.27	0.33	0.12	4.71
4D	121	4.21	0.79	0.31	0.07	0.26	0.31	0.12	4.21
5D	267	2.11	0.81	0.29	0.05	0.24	0.26	0.12	3.09
6D	287	1.65	0.79	0.31	0.06	0.25	0.31	0.12	4.27
7D	235	2.71	0.76	0.32	0.05	0.26	0.32	0.12	4.48
Genome A	3022	1.63	0.80	0.28	0.04	0.24	0.29	0.12	3.89
Genome B	3218	1.61	0.79	0.30	0.04	0.24	0.30	0.12	4.02
Genome D	1686	2.34	0.77	0.32	0.06	0.26	0.32	0.12	4.50
Whole genome	7926	1.77	0.79	0.30	0.04	0.24	0.30	0.12	4.08

Summary statistics of various genetic diversity estimates for each genome of 2,023 wheat landraces had similar values (Table 1). The gene diversity (Hs) in this study ranged from 0.10 to 0.5 with the lowest mean in chromosome 3A (0.23) and highest in chromosome 6A (0.36). Among the three genomes, the B genome showed the highest mean diversity (0.32). The Hs with a value above 0.4 was observed in maximum number of markers (28.24%) and observed least for the value less than 0.1 (2.03%). The PIC was observed to range from 0.09 to 0.38. The mean PIC value on each chromosome showed a similar tend with Hs which ranged from 0.20 (3A) to 0.29 (6A). At the genome level, both A (0.23) and B (0.24) genomes were lower than the D genome (0.26). The MAF from 0.3 to 0.5 was observed in 25.18% of the markers, whereas, MAF less than 0.1 was observed in 29.35%.

The observed nucleotide diversity or average pairwise divergence (π/bp) ranged from 0.23 (7A) to 0.35 (3A) with an average of 0.30. Expected nucleotide diversity or expected number of polymorphic sites (θ/bp) were similar with an average of 0.12. Tajima’s D ranged from 3.89 (A) to 4.50 (D) with an average of 4.08. This value showed significant deviation from the neutral evolution (D = 0) which means the population may have gone through balancing selection. A positive value of D also indicates that rare alleles were present at low frequencies in the population.

### Population structure of the landrace accessions

**Fig. 3:**
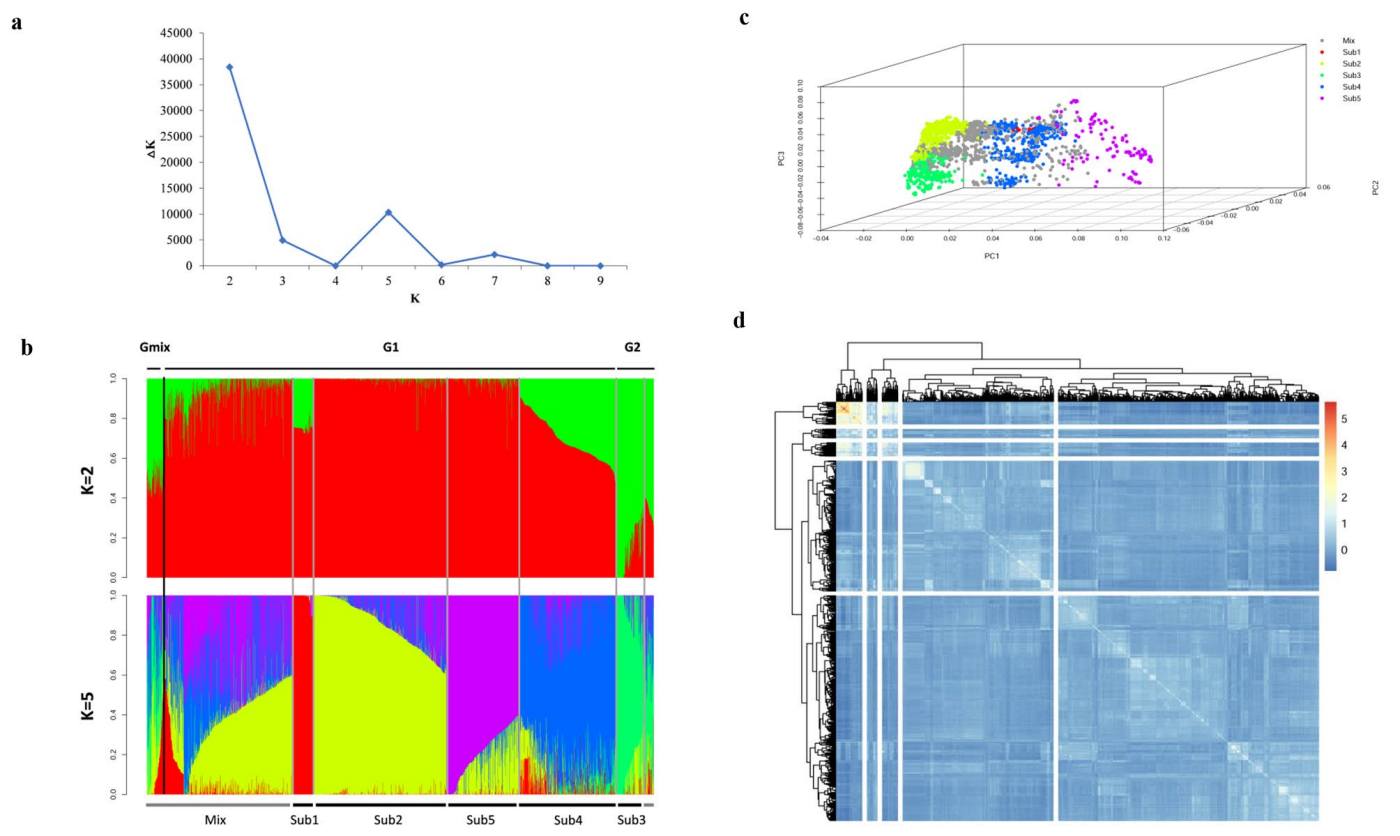
Representation of genetic structure of 2,023 landraces based on population structure analysis and principal component analysis (PCA). a Estimated ΔK of 2,023 landraces over three runs for each K value. b Estimated population structure in 2,023 landraces assessed by STRUCUTRE software. Each individual is represented by a thin vertical bar, partitioned into up to K colored segments. Sub1, Sub2, Sub3, Sub4, Sub5 and Mix are subgroups identified by STRUCTURE assigned with the maximum membership probability. c Display of PCA accessions colored by population subgroups. The different colored pots represent the different subgroups inferred by STRUCTURE analysis. d Heatmaps of kinship matrix based on the 7,962 SNP markers

The population structure of the 2,023 accessions was analyzed using 7,963 high-quality SNPs. STRUCTURE software identified the number of subpopulations. The number of cluster (K) was plotted against ΔK to determine the optimum number of subpopulations. The largest ΔK value was observed at K = 2 suggesting the presence of two main groups (Fig. 3a). The percentage of the membership of each accession in the two groups was presented in Additional file 4: Table S3. When using a probability of membership threshold of 60%, 1,726 and 184 accessions were respectively assigned into subgroups G1 and G2 and the remaining 76 accessions were placed in a mixed subgroup (Gmix) (Fig. 3b). The main groups were further subdivided into Sub1, Sub2, Sub3, Sub4 and Sub5 subpopulations (Fig. 3b). The Sub1 subpopulation included 84 accessions (38.10% from Jiangsu and 16.67% from Sichuan); Sub2 included 528 accessions (39.02% from Jiangsu, 21.02% from Zhejiang, and 10.98% from Shanghai); 115 accessions were in Sub3 (40.00% from Jiangsu and 11.30% from Guizhou); Sub4 included 387 accessions (23.00% from Jiangsu, 19.64% from Henan, 18.60% from Sichuan and 16.80% from Guizhou); Sub5 included 292 accessions, almost 92.47% were from Jiangsu. The remaining 617 accessions, accounting for 30.50% of all germplasm, were classified Mix as they had membership probabilities lower than 0.60 for any given subgroup (Additional file 5: Table S4).

PCA based on 2,228 SNP molecular markers showed a similar, five-cluster distribution pattern, with the mixed subgroup being in the middle of the five defined subgroups (Fig. 3c). In scatterplots, the first three principal components explained 29.36, 12.09 and 7.74% of the total variation, respectively. Overall, five clusters were clearly identified by PCA, in agreement with the results from STRUCTURE. We also calculated a kinship analysis to examine genetic clustering among the landraces, and a heat map was generated on their kinship relationship values using R package (Additional file 6: Table S5). Analysis of kinship indicated five clusters with most accessions (blue) having closely familial relationships (Fig. 3d).

### Genetic differentiation of populations

F-statistics was calculated from 1,406 accessions after removing the 617 Mix population. Binary allelic data per locus was used for statistical analysis and more than 1.3 alleles were effective except for the Sub1 population. As expected, the heterozygosity (H_E_) and Shannon’s diversity index (I) were the most discriminatory measures of differences among the five subgroups, with average genetic diversity estimated to be 0.20 and 0.31 for H_E_ and the I, respectively (Table 2). Sub3 showed the highest genetic variability (H_E_ = 0.36; I = 0.53), whereas Sub1 showed the lowest (H_E_ = 0.05; I = 0.09). The inbreeding coefficients (F) for Sub2, Sub3, Sub4 and Sub5 were > 0.7 whereas that for Sub1 was considerably lower (0.30). Comparing the value of the H_O_ in each subpopulation, Sub1 exhibited the lowest H_O_ value.

**Table 2:** Diversity based on SNPs among the five subgroups

Subgroup	Number of accessions	Na	Ne	I	H_O_	H_E_	F
Sub1	84	1.33	1.08	0.09	0.04	0.05	0.30
Sub2	528	1.92	1.30	0.29	0.04	0.19	0.74
Sub3	115	2.00	1.63	0.53	0.05	0.36	0.85
Sub4	387	1.99	1.45	0.42	0.04	0.27	0.84
Sub5	292	1.99	1.45	0.42	0.04	0.27	0.84
Total	1406	1.85	1.34	0.31	0.04	0.20	0.73

Na, number of different alleles; Ne, number of effective alleles; I, Shannon’s diversity index; H_O_, observed heterozygosity; H_E_, expected heterozygosity; F, inbreeding coefficient

Analysis of the fixation index (F_ST_) values, a measure of genetic differentiation between populations, revealed that the highest genetic differentiation was between Sub1 and Sub3 (F_ST_=0.47), and the slightest difference was between Sub2 and Sub5 (0.11) (Table 3). The Sub3 subpopulation showed the most significant genetic differentiation from other subpopulations.

**Table 3:** F_ST_ values between subpopulations assessed with SNP markers

	Sub1	Sub2	Sub3	Sub4	Sub5
Sub1	-				
Sub2	0.22	-			
Sub3	0.47	0.45	-		
Sub4	0.19	0.13	0.35	-	
Sub5	0.27	0.11	0.44	0.15	-

AMOVA based on the pairwise genetic distances using GenAlEx 6.51b2. AMOVA revealed that 32.84% of the total variation was explained by the differences among the populations, whereas 67.16% of the variation was within the populations (Table 4). This confirmed much greater variation within than between subpopulations.

**Table 4:** The analysis of molecular variance (AMOVA) using 7,963 SNPs and the genetic differentiation among the five subpopulations of the 1,406 wheat landraces

Source of variation	Degrees of freedom (df)	Sum of squares (SS)	Mean sum of squares (MS)	Estimated variance	Percentage of variation (%)	*P* value
Among pops	4	1,740,106.27	435,026.57	1,682.38	32.84	1e-03
Within pops	1,401	4,820,898.24	3,441.04	3,441.04	67.16	
Total	1,405	6,561,004.51		5,123.42	1	
Nm	0.51					

### Core collection

**Fig. 4:**
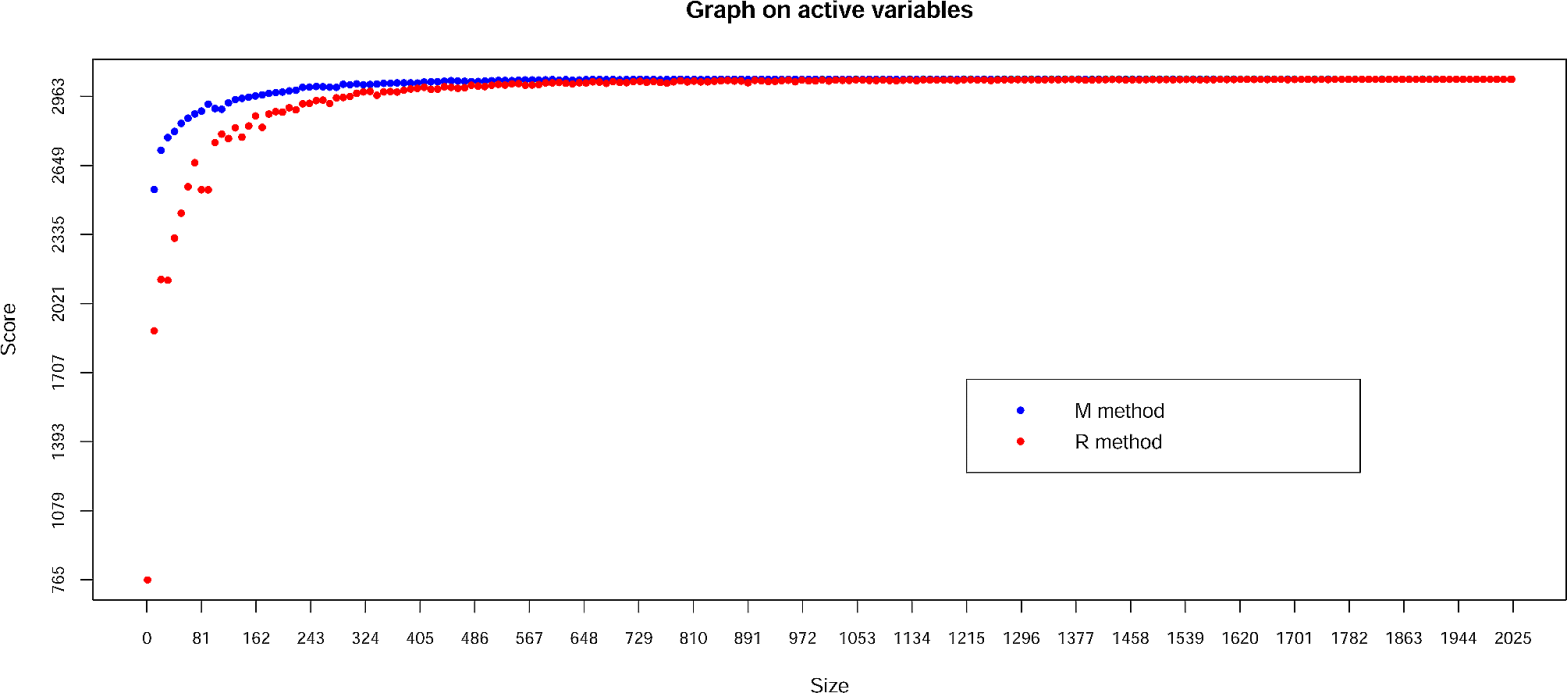
Prediction of core collection sample size by maximization (M) method and random (R) method

Maximization (M) and Random (R) algorithm methods were used to predict the optimal sample size of the core germplasm (Fig. 4). The M score was higher than the R score, regardless of the sample size change, indicating that the M method of sampling alleles was significantly more efficient than the R method. When 304 accessions were selected, the M curve nearly reached a plateau (score = 3,041), indicating that 304 accessions (15.0%) were more suitable to define the core collection. We used the M method to extract 51, 102, 203, 304, 405 and 506 samples; these six sample sizes of the core collection captured 2.5, 5.0, 10.0, 15.0, 20.0 and 25.0% of the raw materials, respectively (Table 5).

**Table 5:** Nested core collection sample size predicted by maximization (M) method and random (R) method

Core collection	Size	Major allele frequency	Genotype Number	Gene diversity	Heterozygosity	PIC	Distribution in groups					
							Sub1	Sub2	Sub3	Sub4	Sub5	Mix
CC51	51	0.76	2.93	0.34	0.11	0.27	2	13	9	12	2	13
CC102	102	0.78	2.95	0.31	0.07	0.26	4	36	12	15	6	29
CC203	203	0.79	2.94	0.30	0.05	0.24	9	47	14	37	30	66
CC304	304	0.81	2.94	0.27	0.04	0.22	13	109	10	49	40	83
CC405	405	0.80	2.95	0.28	0.04	0.23	15	109	11	88	57	125
CC506	506	0.80	2.95	0.28	0.04	0.23	24	138	14	104	62	164

**Fig. 5:**
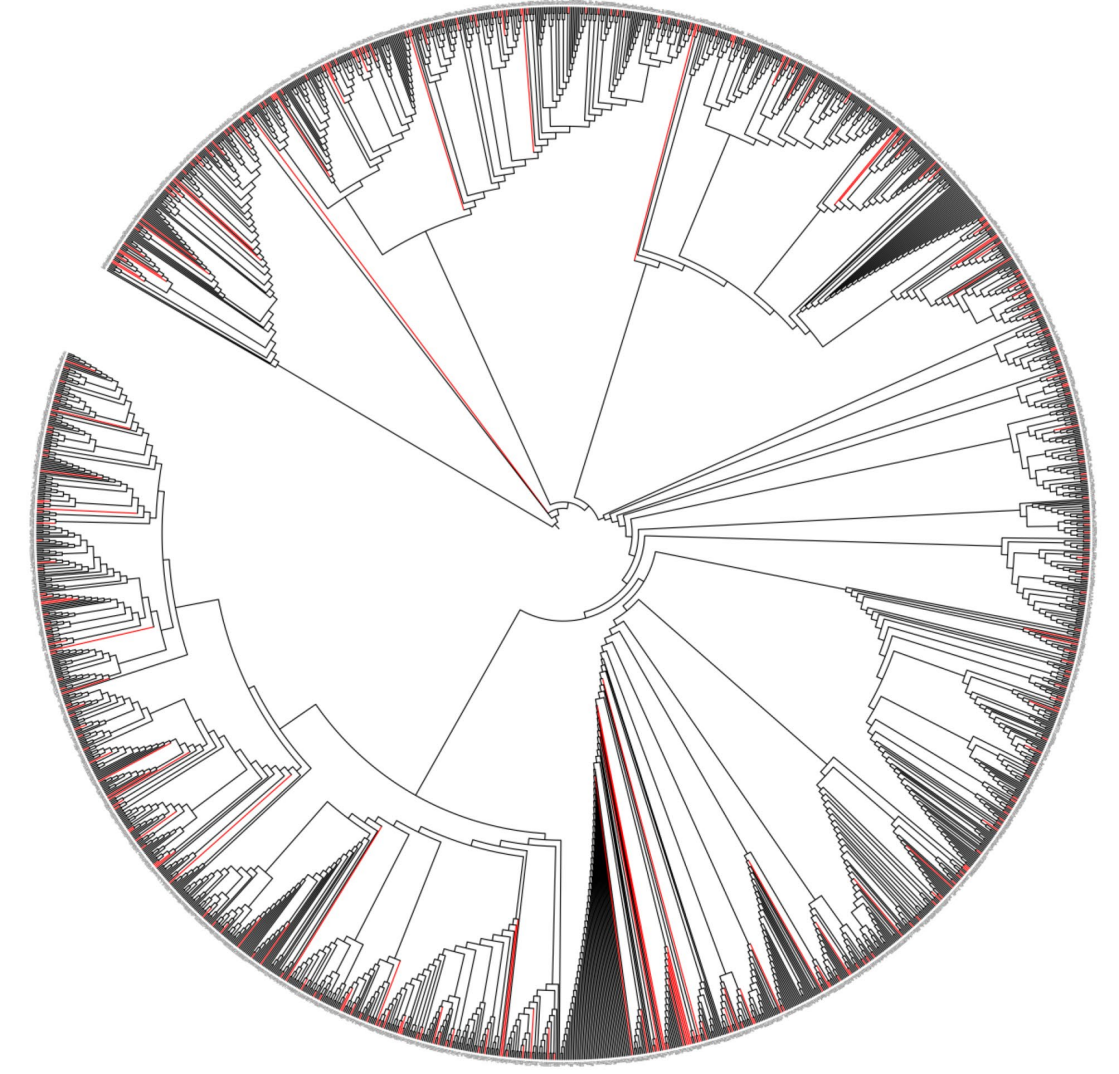
Neighbor-joining clustering of 2,023 bread wheat landrace accessions. Red lines represent the core collection, and black lines represent the original wheat landrace collection

Considering some landrace accessions with outstanding disease resistance, 311 accessions, accounting for 15.37% of the original set, formed the final core collection of (Fig. 1). Among them, 13 accessions were from Sub1, 81 were from Sub2, 17 were from Sub3, 60 were from Sub4, 45 were from Sub5, and 95 were from Mix. The genetic diversity index and PIC values were 0.31 and 0.25, respectively, and higher than those of full collection (0.30 and 0.24) (Table 6). The neighbor-joining tree constructed with the 7,926 SNP markers showed that the final primary core accessions were evenly distributed among the original collection and were highly representative (Fig. 5 and Additional file 7: Table S6). After accounting for uniformity and redundancy in the agronomic traits, we finally selected 311 accessions as the core collection. A comparison of diversity indices (${H}^{{\prime }}$) between the full landrace collection and the 311-core collection showed no significant differences at 12 agronomic traits (Table 7).

**Table 6:** Comparison of number of alleles, gene diversity and polymorphism information content (PIC) between the 2,023 landraces and core accessions subgroups at the genome level

Subgroup	No. of alleles			Gene Diversity		PIC	
	All	Core accessions	Sample %	All	Core accessions	All	Core accessions
Sub1	10,498	10,243	97.57	0.05	0.09	0.04	0.07
Sub2	15,124	14,253	94.24	0.19	0.19	0.15	0.15
Sub3	15,831	15,175	95.86	0.36	0.34	0.28	0.27
Sub4	15,794	15,653	99.11	0.27	0.28	0.22	0.23
Sub5	14,696	13,941	94.86	0.20	0.21	0.16	0.17
Mix	15,852	15,852	100	0.30	0.31	0.24	0.25
Total	15,852	15,852	100	0.30	0.31	0.24	0.25

**Table 7:** Comparison of genetic diversity index (${H}^{{\prime }}$) between the 2,023 landraces and core accessions at the phenotypic level

Categories	Trait name	Group	$H{\prime }$	t-value	*p*-value
Plant morphological traits	Awn type	All	0.82	-0.52	0.63
		Core accessions	0.84		
	Glume color	All	0.64	-1.28	0.42
		Core accessions	0.66		
	Spike type	All	0.92	-1.69	0.19
		Core accessions	0.99		
	Plant height	All	2.04	0.21	0.84
		Core accessions	2.03		
	Spike length	All	2.09	-0.04	0.97
		Core accessions	2.09		
Maturity traits	Heading date	All	1.94	-0.18	0.86
		Core accessions	1.96		
	Flowering date	All	2.00	-0.36	0.73
		Core accessions	2.03		
Yield-related traits	Spikelet number per spike	All	2.06	-0.13	0.90
		Core accessions	2.07		
	Sterile spikelet number per spike	All	2.04	-0.55	0.60
		Core accessions	2.09		
	Grain number per spikelet	All	1.97	0.30	0.54
		Core accessions	1.95		
	Grain number per spike	All	2.06	0.64	0.54
		Core accessions	2.04		
	Thousand kernel weight	All	2.06	-0.81	0.44
		Core accessions	2.10		

## Discussion

Diversity among landraces was initially described using spike morphology traits and botanical variety classification [14, 33]. Some landraces were mixtures of different wheat morphotypes that were easily identified by spike color or awn features. Landraces with the same name but originating from different regions often had different phenotypes. Likewise, landraces with similar morphotype had different origin and names. With this study we gained insights into the genetic diversity of landraces accessions preserved in the wheat collection at Jiangsu Academy of Agricultural Sciences. Although the yield of landraces is generally less than that of commercial varieties grown under current agronomic conditions, they remain important sources of genetic variation in searching for novel sources of resistance to biotic and abiotic stress [34]. For example, Chinese landraces such as Wangshuibai, Haiyanzhong, Baisanyuehuang and Huangfangzhu from Jiangsu province have high levels of resistance to Fusarium head blight (FHB) resistance and have been used as donor sources in breeding [35–38].

### Genetic diversity of landrace accessions

Evaluation of genetic diversity in germplasm resources is of great significance for conservation, breeding and research. Studies have repeatedly documented much higher genetic diversity in landraces than among elite cultivars [6, 39]. A study by Sansaloni et al. [40] revealed landraces with unexplored diversity and genetic footprints left by selection in different geographical regions; indeed, very little of the genetic diversity had been used in modern breeding. This was also confirmed by analysis of the collection assembled by Watkins in the early 1900s [41, 42]. Selection in modern breeding programs has led to decreased genetic diversity in current wheat populations, and unless diversity can be maintained in gene banks, it will be lost for future generations [43]. Thus, landraces may hold novel variability not present in modern elite cultivars [17, 44, 45].

In the present study, 7,926 high quality SNPs and 12 phenotypic data of related traits obtained from 2,023 Chinese landrace accessions were used. A large portion of the polymorphic markers were mapped to the B genome (40.60%), followed by the A genome (38.13%) and the D genome (21.27%) (Fig. 2), which was in agreement with previous studies [46]. Interestingly, the Hs, PIC and π on the D genome was higher than the A and B genome in this study (Table 1). Generally, the D genome was the least diverse genome in previous studies [21, 47]. The greater diversity of the D genome in Chinese landrace accessions may indicate a greater possibility that the D genome has novel genetic variations [48], which can be used in elite wheat breeding programs to reduce the bottleneck of the D genome and broaden the genetic base [49].

### Population structure and relationship

The population structure analysis is the first step in conducting the association mapping studies. In the present study, STRUCTURE, PCA and kinship analysis showed that there are most probably five subpopulations in the studied collection of landrace accessions (Fig. 3). In each subpopulation, there were genotypes from different regions (Additional file 8: Fig. S2a and b). A few accessions showed a certain association between geographical origin and population structure (Additional file 8: Fig. S2c, d and e). This is a common phenomenon for most cereal landraces worldwide because of informal seed exchange systems involving regional and countrywide farming communities [50, 51]. Nearly 30.50% of landrace accessions were classified into Mix subpopulations, which may also be indirectly attributed to the continuous gene flow of landrace genotypes among the different regions.

Genetic differentiation among populations is reflected by F_ST_ [52, 53]. F_ST_ measures population differentiation due to genetic structure and a value greater than 0.15 predicts significant genetic differentiation between subpopulations [54]. High genetic differentiation among subpopulations is indicative of a low level of gene flow between subpopulations. For example, a low level of gene flow was also reported among the wheat landrace populations of Mediterranean origin [55]. This phenomenon may be due to deploying newly developed cultivars across multiple countries and less using of old wheat landraces and locally selected germplasm in breeding programs [56]. AMOVA indicated that most of the genetic variation (67.16%) occurred within subpopulations, confirming the existence of considerable unique variation in subpopulations (Table 4). Previous studies have reported similar results, but it is still unclear whether genetic variation within subpopulations is due to variations that occurred during different domestication processes or introduced by farmers and traders from other regions [56]. In this study, 30.50% of landrace accessions were classified as Mix in population structure analysis, which may be attributed to germplasm exchange between different regions.

### A core collection of wheat landraces

A core collection that represents the genetic diversity of a crop in a minimal number of accessions is an effective way to achieve efficient conservation and utilization of germplasm [57, 58]. Ideally, a core collection should be approximately 10% of the total collection and retain 70% of the genetic diversity from the initial collections [59]. The number of accessions selected for the core collection depends on the size of the initial collection and the sampling ratio [38]. Li et al. [60] proposed sampling 5–40% of accessions to construct core germplasm, with 10% being optimum. Van Treuren et al. [61] developed an advanced cultivar core collection of bare cultivars using a sampling percentage of 26.92%. Hao et al. [20] constructed a mini-core collection, accounting for 5% of the initial collection and representing 91.5% of the genetic diversity of the initial collection. Xu et al. [57] suggested that a sampling percentage of 20% was an appropriate size to construct a core collection for barely. In this study, we selected 15.37% (311/2023) of accessions as our core collection.

Representative core accessions have been selected in diverse crops using various sampling strategies and clustering methods [20, 62–64]. Previous studies indicated that M strategy performs well when accessions come from populations with restricted gene flow or are from self-pollinated species [57, 65, 66]. The MSTRAT algorithm is one of the representative core selection methods for implementing the M strategy [32]. Here, we used the M strategy as implemented in MStratv 4.1 software and successfully established a representative core collection with high genetic diversity.

Using genotypic and phenotypic information along with clustering to construct a core collection is more efficient than using genotypic or phenotypic information alone [65]. It is important to verify the quality of a core collection, as the quality determines the direction of subsequent research [67]. In the present study, the genetic diversity indices (${H}^{{\prime }}$) of 12 morphological characters in the core collection was not significantly different from the entire collection, indicating that the core collection can effectively represent the variation range of 12 morphological traits of the original set. In general, molecular markers reflect changes in genetic variation at the DNA level, without environmental interference, hence providing valuable data to describe genetic diversity. In this study, the 311 accessions were selected as a core collection of wheat landraces, which retained 100% of alleles in a primary core collection. The genetic diversity and PIC value of the core collection were higher than the initial collection. The combined results indicate that the core collection selected in this study well represents the initial landrace collection.

## Conclusions

Constructing the core collections of wheat landrace will enhance the efficiency of management and utilization of accessions in the germplasm banks. In the present study, we constructed a core collection of 311 accessions representing 100% of the SNPs identified among 2,023 wheat landrace accessions held by the Jiangsu Provincial Crop Germplasm Resource Genebank. The evaluation showed that this core collection is high-quality and valuable for phenotypic and genetic studies. The core collection can be used as a primary germplasm resource for mining novel genes, genetic association and functional gene analyses.

### Electronic supplementary material

Below is the link to the electronic supplementary material.

**Additional file 1:Table S1.** Detailed information of 2,023 wheat landraces accessions.

**Additional file 2:Table S2.** Categories and descriptive statistics for the 12 agronomic traits.

**Additional file 3:Fig. S1.** Distribution and density of filtered single nucleotide polymorphisms (7,963 SNPs) across 21 chromosomes. Horizontal display chromosome length. The number of SNPs in a given region is indicated at the bottom right side.

**Additional file 4:Table S3.** Individual Q matrix calculated in STRUCTURE (K = 2).

**Additional file 5:Table S4.** Individual Q matrix calculated in STRUCTURE (K = 5).

**Additional file 6:Table S5.** The kinship relationships matrix between accessions.

**Additional file 7:Table S6.** Estimates of evolutionary divergence between accessions.

**Additional file 8:Fig. S2.** Grouping of 2,023 wheat landrace accessions by principal component analysis. a-b Plots of PC1, PC2 and PC3 of landrace accessions based on predicted group membership from STRUCTURE (K = 5). b-c Plots of PC1, PC2 and PC3 from principal component analysis of landrace accessions from different regions of China. e Geographic locations of 2,023 wheat landraces based on predicted group membership from STRUCTURE (K = 5).

## Data Availability

The datasets used or analyzed during the current study are available in this published article in the additional files. SNPs data used in this study is availability in the in China National Center for Bioinformation (CNCB) repository under accession number GVM000783 (https://ngdc.cncb.ac.cn/gvm/getProjectDetail?project=GVM000783).

## Abbreviations

AMOVA: Molecular variance analysis
CC: Core collection
df: Degrees of freedom
FHB: Fusarium head blight
F: Inbreeding coefficients
GWAS: Genome-wide association study
H: Heterozygosity
H': Genetic diversity indices
H_E_: Heterozygosity
H_O_: Observed heterozygosity
Hs: Gene diversity
I: Shannon’s diversity index
LD: Linkage disequilibrium
MAF: Minor allele frequency
MAS: Marker-assisted selection
MS: Mean sum of squares
N_a_: Number of different alleles
Ne: Number of effective alleles
PIC: Polymorphism information content
SNP: Single nucleotide polymorphism
SS: Sum of squares
SSR: Simple sequence repeat
θ: Expected nucleotide diversity or estimated mutation rate
π: Average pairwise divergence or observed nucleotide diversity
