# Supplementary figures and images for: Genetic diversity and population structure of wheat landraces in Southern Winter Wheat Region of China
Source: BMC Genomics. 2024 Jul 3;25:664. doi: 10.1186/s12864-024-10564-z (PMC11223385; doi:10.1186/s12864-024-10564-z)

**Fig. S1**
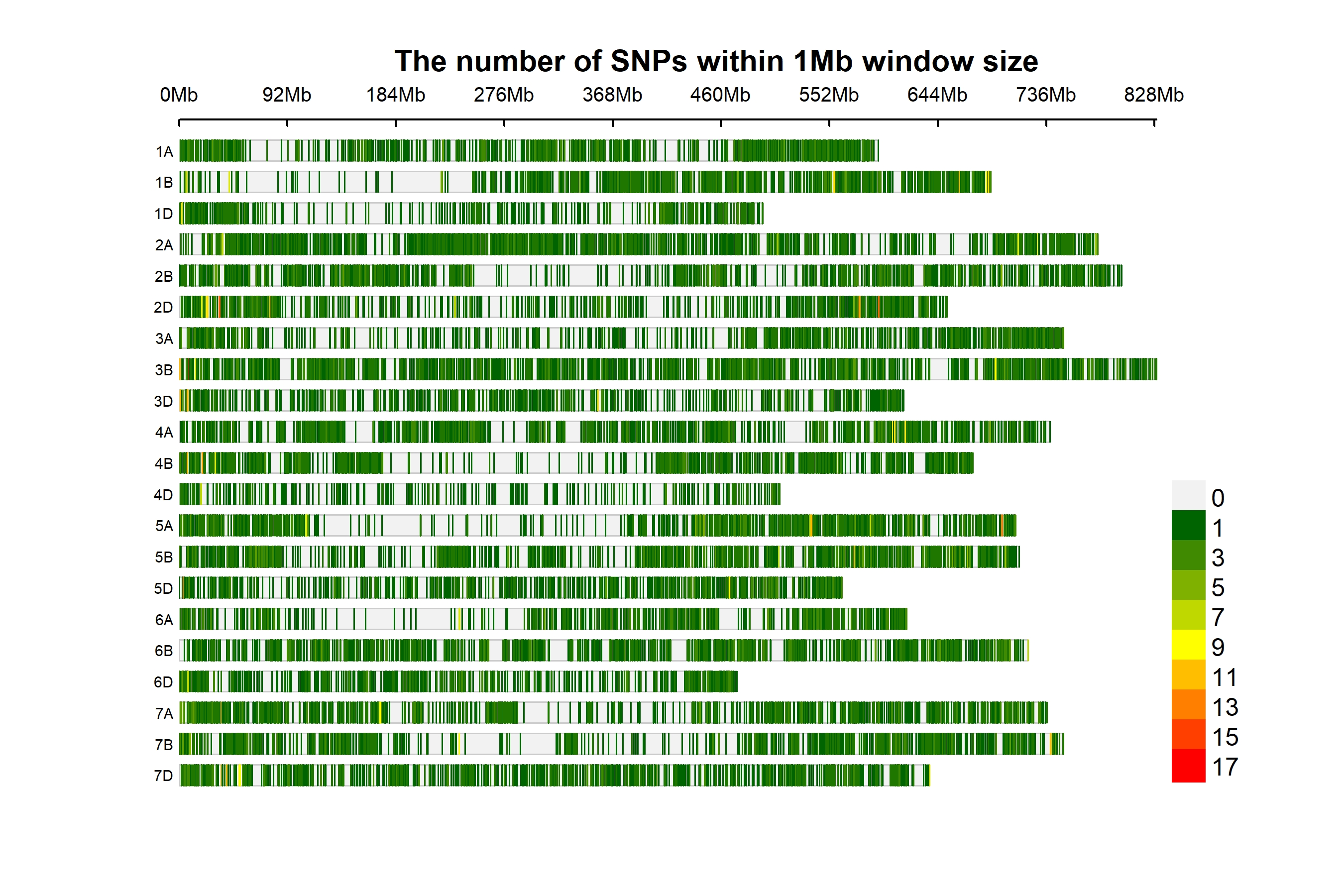

Supplement: Supplementary file 3 — Additional file 3:Fig. S1. Distribution and density of filtered single nucleotide polymorphisms (7,963 SNPs) across 21 chromosomes. Horizontal display chromosome length. The number of SNPs in a given region is indicated at the bottom right side. [file 12864_2024_10564_MOESM3_ESM.docx]

**Fig. S2**


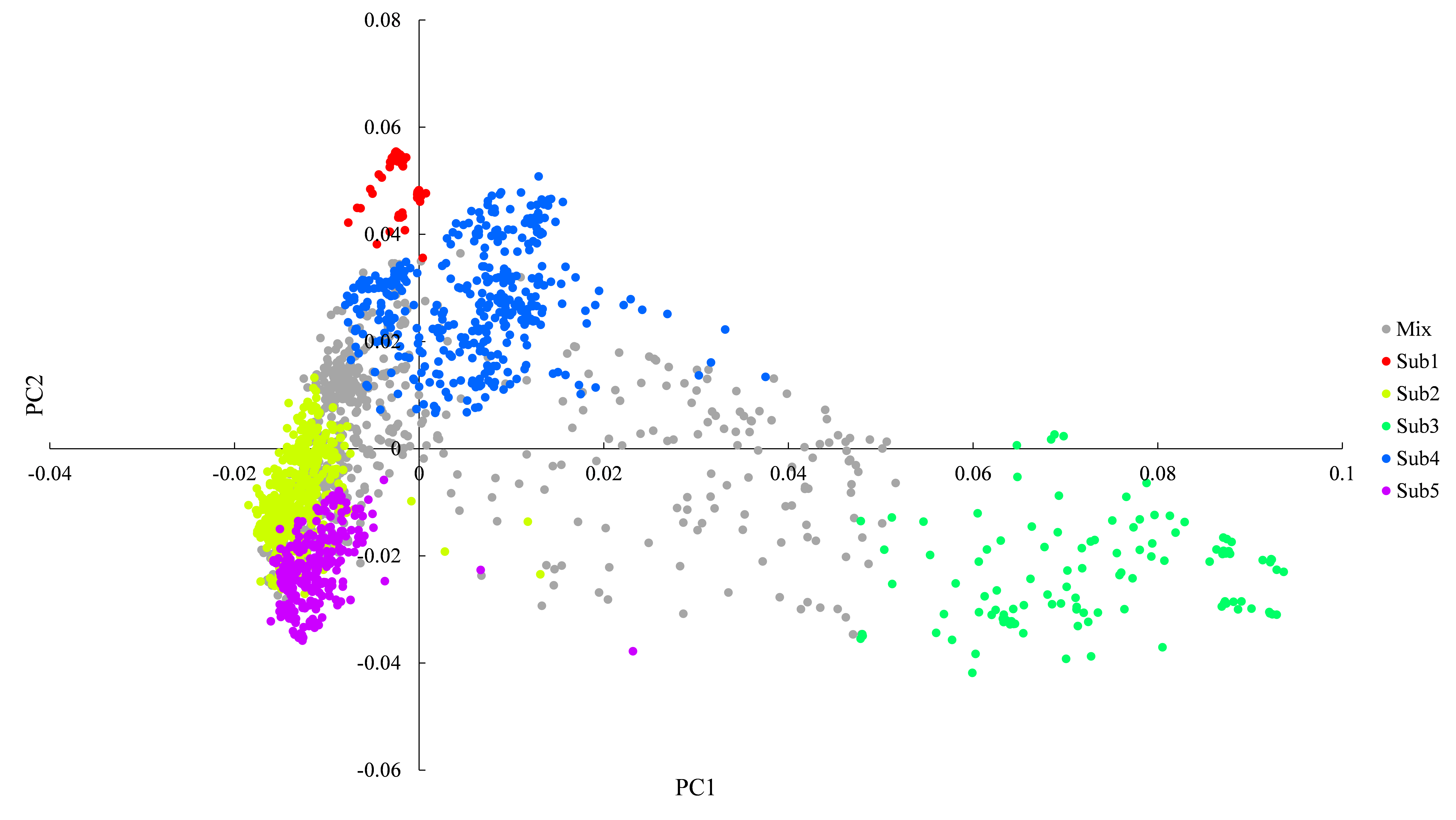


**a**


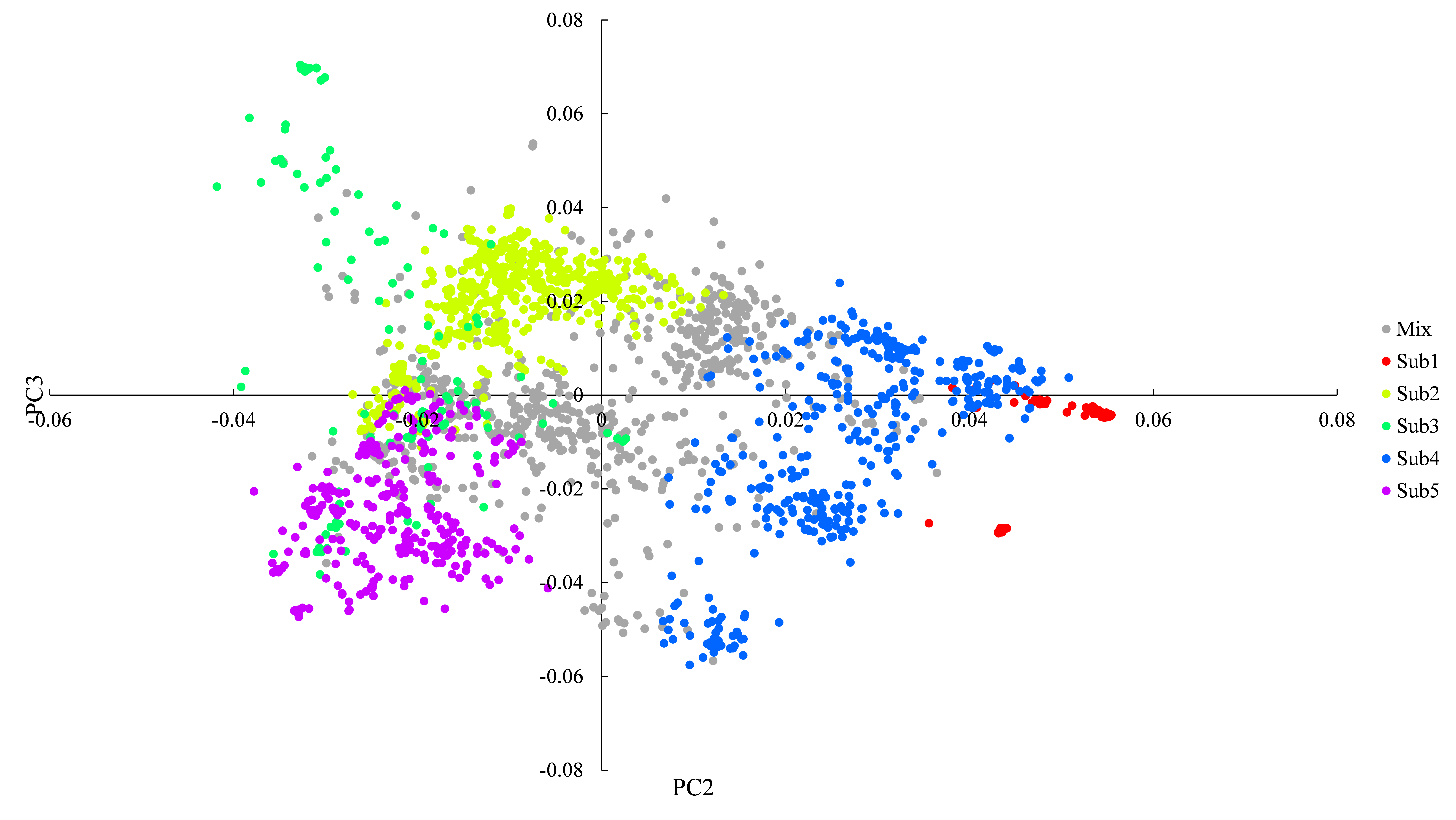


**b**


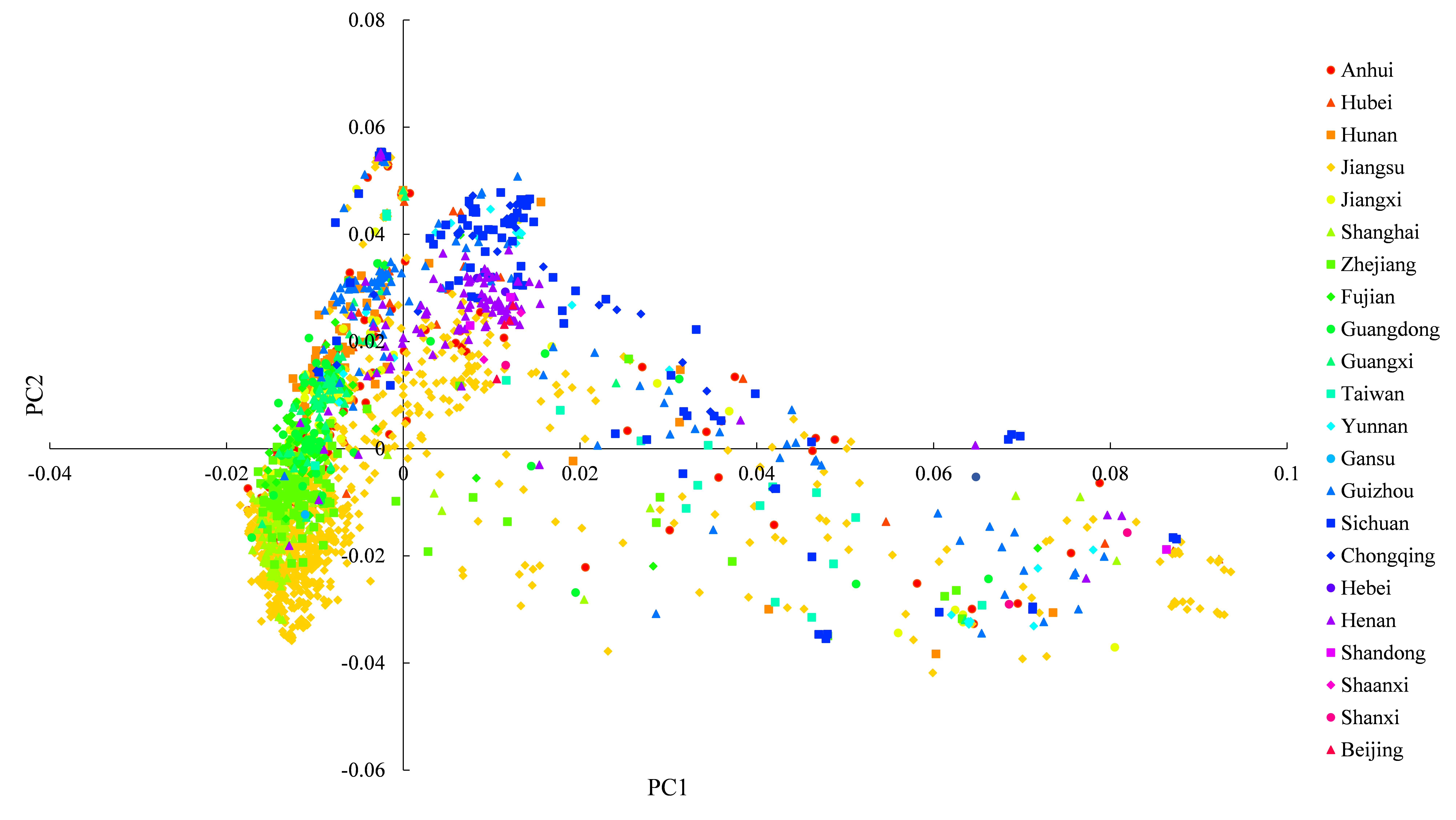


**c**


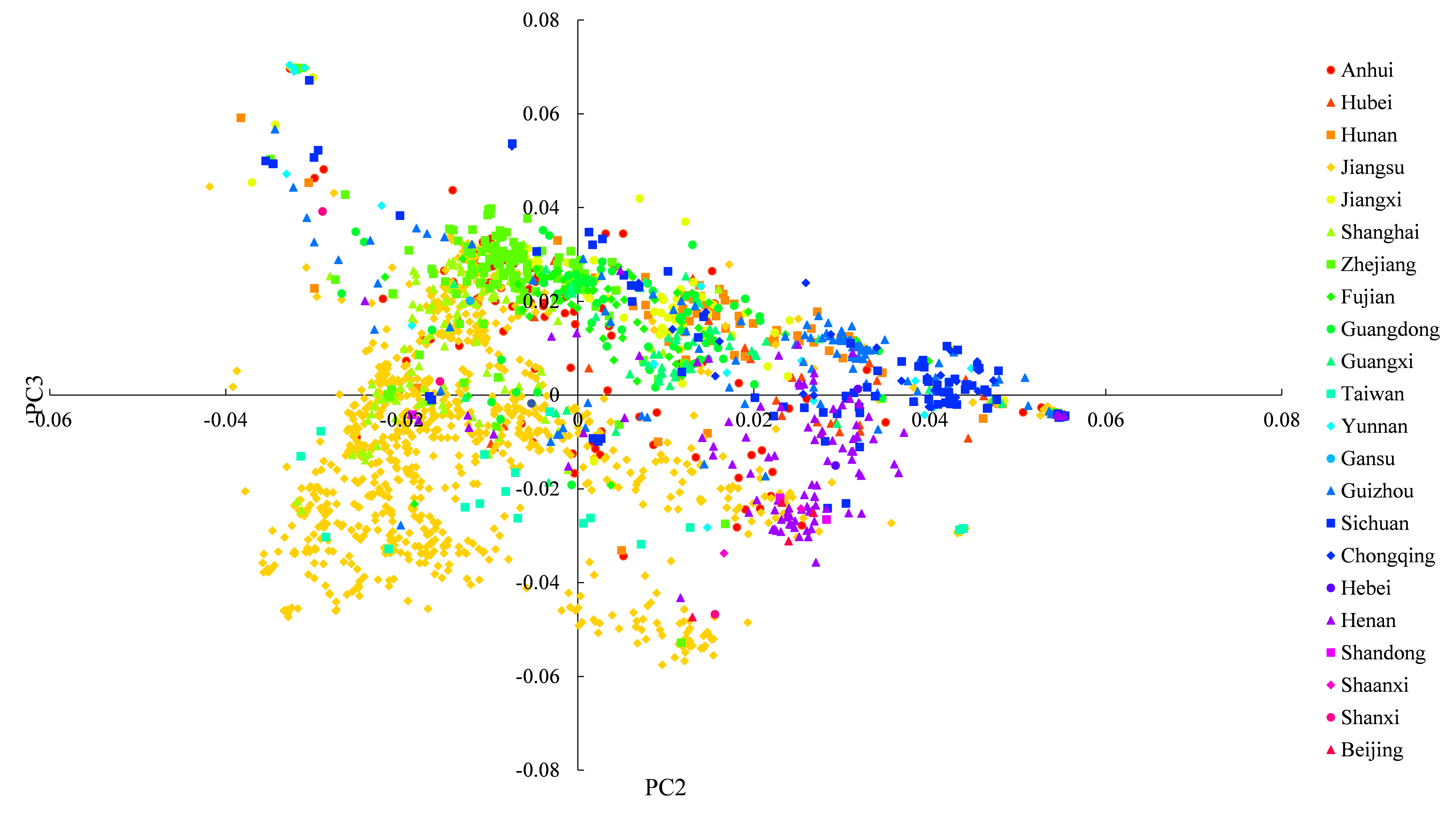


**d**

**e**


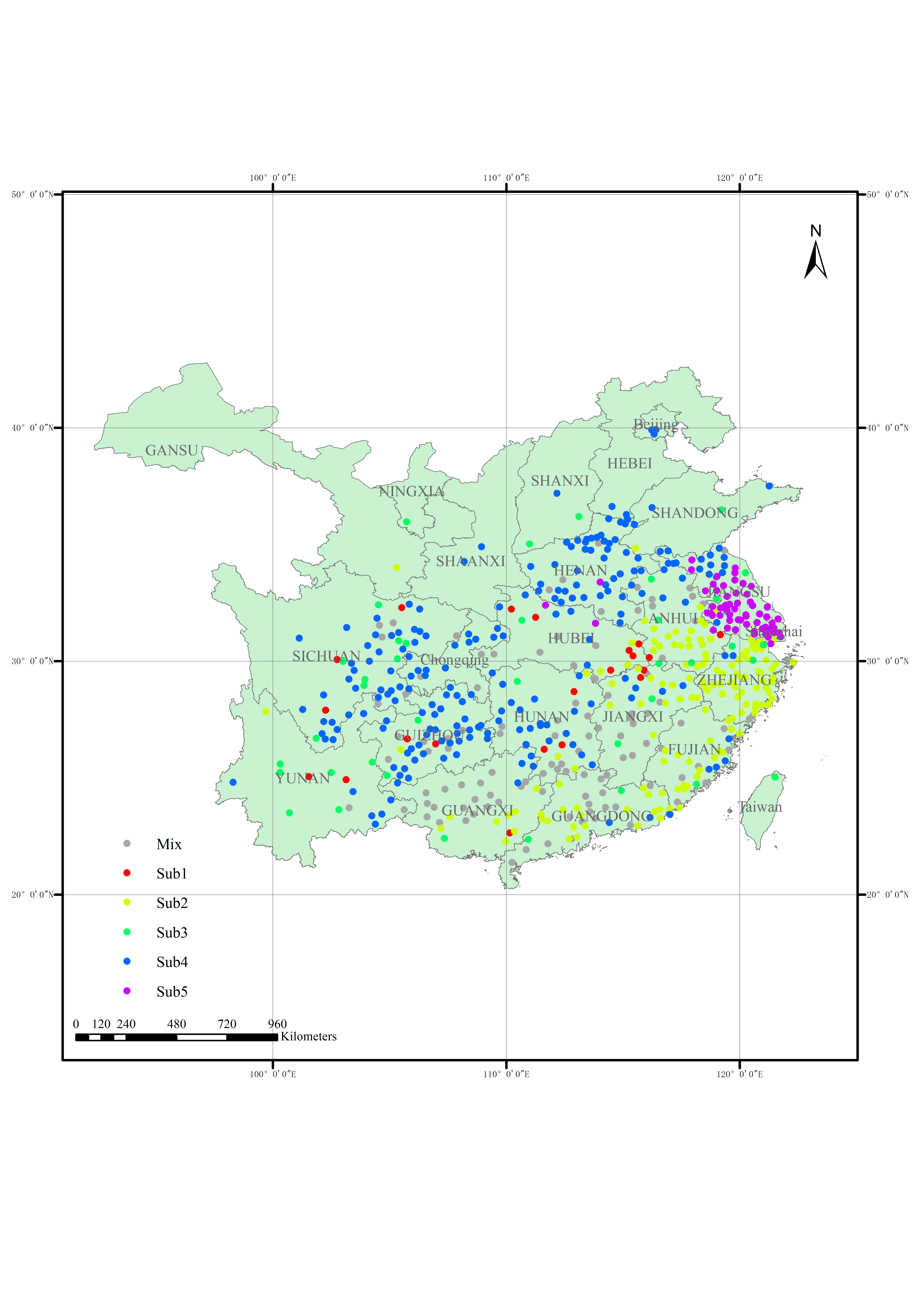

Supplement: Supplementary file 8 — Additional file 8:Fig. S2. Grouping of 2,023 wheat landrace accessions by principal component analysis. a-b Plots of PC1, PC2 and PC3 of landrace accessions based on predicted group membership from STRUCTURE (K = 5). b-c Plots of PC1, PC2 and PC3 from principal component analysis of landrace accessions from different regions of China. e Geographic locations of 2,023 wheat landraces based on predicted group membership from STRUCTURE (K = 5). [file 12864_2024_10564_MOESM8_ESM.docx]
